# A deep learning-based model for detecting *Leishmania* amastigotes in microscopic slides: a new approach to telemedicine

**DOI:** 10.1186/s12879-024-09428-4

**Published:** 2024-06-01

**Authors:** Alireza Sadeghi, Mahdieh Sadeghi, Mahdi Fakhar, Zakaria Zakariaei, Mohammadreza Sadeghi, Reza Bastani

**Affiliations:** 1 Department of Mechatronics Engineering, Faculty of New Sciences and Technologies, University of Tehran, Tehran, Iran; 2 Student Research Committee, Mazandaran University of Medical Sciences, Sari, Iran; 3 Iranian National Registry Center for Lophomoniasis and Toxoplasmosis, Imam Khomeini Hospital, Mazandaran University of Medical Sciences, P. O box, Sari, 48166-33131 Iran; 4 Toxicology and Forensic Medicine Division, Mazandaran Registry Center for Opioids Poisoning, Anti-microbial Resistance Research Center, Imam Khomeini Hospital, Mazandaran University of Medical Sciences, Sari, Iran; 5 Student Research Committee, Sari Branch, Islamic Azad University, Sari, Iran

**Keywords:** Leishmania, Deep learning, Transfer learning, Image processing, Machine learning, Artificial intelligence, Microscopic images

## Abstract

**Background:**

Leishmaniasis, an illness caused by protozoa, accounts for a substantial number of human fatalities globally, thereby emerging as one of the most fatal parasitic diseases. The conventional methods employed for detecting the *Leishmania* parasite through microscopy are not only time-consuming but also susceptible to errors. Therefore, the main objective of this study is to develop a model based on deep learning, a subfield of artificial intelligence, that could facilitate automated diagnosis of leishmaniasis.

**Methods:**

In this research, we introduce LeishFuNet, a deep learning framework designed for detecting *Leishmania* parasites in microscopic images. To enhance the performance of our model through same-domain transfer learning, we initially train four distinct models: VGG19, ResNet50, MobileNetV2, and DenseNet 169 on a dataset related to another infectious disease, COVID-19. These trained models are then utilized as new pre-trained models and fine-tuned on a set of 292 self-collected high-resolution microscopic images, consisting of 138 positive cases and 154 negative cases. The final prediction is generated through the fusion of information analyzed by these pre-trained models. Grad-CAM, an explainable artificial intelligence technique, is implemented to demonstrate the model’s interpretability.

**Results:**

The final results of utilizing our model for detecting amastigotes in microscopic images are as follows: accuracy of 98.95 1.4%, specificity of 98 2.67%, sensitivity of 100%, precision of 97.91 2.77%, F1-score of 98.92 1.43%, and Area Under Receiver Operating Characteristic Curve of 99 1.33.

**Conclusion:**

The newly devised system is precise, swift, user-friendly, and economical, thus indicating the potential of deep learning as a substitute for the prevailing leishmanial diagnostic techniques.

## Background

Neglected tropical diseases (NTDs) that affect about 1.7 billion people in tropical countries are a group of 20 diseases, including leishmaniasis [1], which is the main focus of this article. It seems that NTDs are largely related to socioeconomic conditions and they are more prevalent in tropical countries with low income, the causes of which can be pointed to the impact of people’s poverty in limiting their access to health care, education, housing, and proper nutrition, which causes the continuation of this cycle of disease and poverty [2, 3]. Despite numerous efforts to eradicate these diseases, according to reports, their incidence has increased between 2006 and 2016 [4]. On the other hand, during the past few decades, more than half of the forest areas have been destroyed in the direction of the development of the urban industry and agricultural pastures [5]. These changes along with the recent climate changes lead to the expansion of suitable habitats for the vectors and reservoirs of these diseases [6], all of which can create a significant challenge in public health shortly.

Leishmaniasis, which is a zoonotic disease, is caused by an intracellular single-celled parasite called *Leishmania*. It has a complex life cycle that alternates between the vector and the host. Its vector is the phlebotomus female mosquito, which injects flagellated promastigotes (motile form) into the host’s body with its bite while feeding on their blood, and these promastigotes become amastigotes (non-motile form) in the host’s body [7]. Their hosts are often vertebrate mammals such as domestic animals, sylvatic, and humans [8, 9]. *Leishmania* parasites include about 20 species, which, based on the type of pathogen and the host’s immune response, lead to different forms of this disease, and finally, in a general classification, they are divided into four main categories, including cutaneous leishmaniasis (CL), mucocutaneous leishmaniasis (MCL), visceral leishmaniasis (VL), and post-kala-azar dermal leishmaniasis (PKDL) [10]. The symptoms caused by each of these groups can include a range of mild symptoms, in the form of destructive skin lesions (in CL) that have a psychosocial burden for the patient and affect the quality of life, to severe symptoms, including anemia, weight loss, hepatosplenomegaly, and bleeding, which can lead to death [11, 12].

Despite the introduction of new and advanced methods for diagnosis in recent years, such as advanced molecular techniques, flow cytometry, nanodiagnostics, and proteomics, traditional methods (including molecular, immunological, and parasitological methods) are still the gold standard diagnostic method, which is based on microscopic observation of stained culture of tissue or parasite to look for Leishman-Donovan bodies [13, 14], but its specificity and sensitivity are limited because it depends on the parasite load and the operator’s skills [15]. Currently, the treatment includes antimonials, miltefosine, pentamidine, amphotericin B, paromomycin, and combined treatment with these medications, and the mentioned treatments can lead to problems such as serious complications in some patients, drug resistance, and toxicity [16]. Because the treatment of these patients is somewhat challenging and vital, as well as the undeniable role of failure and delay in diagnosis in increasing the mortality rate in endemic areas, it is obvious that the attention of researchers is directed toward the correct and timely diagnosis of the disease. Accurate diagnosis of leishmaniasis requires the identification of the parasite in tissue samples, which is typically done by microscopic examination. However, microscopy can be time-consuming and requires specialized expertise and adequate facilities. However, these resources are not accessible in prospective endemic regions.

Telemedicine, the use of telecommunication and information technologies to provide healthcare services remotely [17], can have a noteworthy impact on managing CL outbreaks. A crucial aspect of intelligent telemedicine is the integration of machine learning and deep learning techniques, which have seen extensive application in medical research in recent years. While these techniques have been widely used for diagnosing protozoan parasites like *Plasmodium* [18–20] and *Trypanosoma* [21, 22], their application in diagnosing leishmaniasis has been comparatively limited. Gorriz et al. [23] developed U-Net [24] using 45 self-collected leishmaniasis microscopic images to segment them and classify different segments into various objects like promastigote and amastigote. Arce-Lopera et al. [25], utilized transfer learning by fine-tuning the VGG19 model [26] with a dataset of 2022 images of CL, melanoma, and other diseases frequently mistaken for CL. Larios et al. [27], employed algorithms such as Principal Component Analysis (PCA), K-Nearest Neighbor (KNN), Support Vector Machine (SVM), and discriminant analysis to classify blood samples from 48 dogs, 20 of which tested positive for *Leishmania*. Mainye et al. [28] trained a deep model, ResNet18 [29], using 401 microscopic images from blood samples of 15 mice to classify images into *Leishmania*, *Trypanosoma*, and *Plasmodium* categories. Zare et al. [30], presented a *Leishmania* parasite detection system based on the analysis of 300 self-collected images from 50 slides, 25 of which were positive for *Leishmania*. This system utilized the AdaBoost technique for final image classification after extracting crucial features from the input data.

In this study, we introduce LeishFuNet, a deep learning model designed for the detection of *Leishmania* patients from their microscopic images. Leveraging transfer learning, specifically employing a feature fusion technique known to be beneficial for models trained on small-sized datasets [31–33], our model demonstrates promising capabilities in this domain. The key contributions of our research are as follows:

- **Development of LeishFuNet**: We developed LeishFuNet, a deep learning model to detect *Leishmania* in a patient-level analysis (i.e., identifying *Leishmania -*infected patients). To our knowledge, this is the first study to undertake such research, as previous studies mainly focused on slide-level analysis (i.e., detecting *Leishmania* slides, where each patient can contribute multiple slides to the dataset).
- **Introduction of a Novel Public Dataset**: We offer a novel and publicly accessible dataset comprising *Leishmania* microscopic images. A significant challenge in the field of developing deep learning techniques for *Leishmania* -related studies is the limited accessibility to public datasets, as most previous studies relied on private datasets and did not share their data publicly. Our new dataset can assist researchers in the field in developing new techniques for *Leishmania* detection.
- **Implementation of Grad-CAM for Model Interpretability**: We integrated Grad-CAM, an explainable artificial intelligence (XAI) technique, to enhance the interpretability and trustworthiness of our model. This study stands out as one of the pioneering efforts to apply XAI techniques to deep learning models developed for *Leishmania* detection. By incorporating Grad-CAM, we ensure that our model’s predictions align closely with those of clinicians, thereby enhancing its reliability for practical use.

In the subsequent sections of this paper, we begin by presenting detailed information about the dataset introduced in this study, along with the preprocessing steps undertaken, and an overview of the LeishFuNet architecture in the ‘Materials and Methods’ section. Following this, in the ‘Results’ section, we present the outcomes obtained by evaluating the proposed model on both our dataset and a related dataset. Additionally, we include the results of implementing Grad-CAM on the LeishFuNet predictions. Moving on to the ‘Discussion’ section, we elaborate on and compare our proposed model with existing models, assessing its advantages and limitations. Finally, in the ‘Conclusion’ section, we summarize the study and draw conclusions based on our findings.

## Materials and methods

### Data acquisition

This study involved the examination of 292 patients suspected of having CL, who referred to the Leishmaniasis Regional Diagnostic Laboratory (LRDL), Sari, Mazandaran, northern Iran. Skin scraping samples were obtained from the lesions of these patients and were subsequently stained with Giemsa for the purpose of enhancing the visibility of any *Leishmania* parasites present. Out of the 292 patients, 138 were determined to be positive for leishmaniasis based on the identification of *Leishmania* parasites in their samples, while the remaining 154 were negative. An image of each of the samples were captured using a digital microscope (Olympus-CX23-TR-Japan) at a magnification of 100x.

### Data preprocessing

To prepare the microscopic images for use in our proposed deep learning model, several preprocessing steps were undertaken. Firstly, all images were resized to a standard size of 224 × 224 pixels. This standardization ensures uniformity in the input data for the model, as each image in the raw dataset may initially have had varying dimensions. Secondly, the pixel values of all images were rescaled to fall within the range of 0 to 1. This rescaling was achieved by dividing each pixel’s original value by 255. By doing so, we normalize the pixel intensities, facilitating better convergence and training efficiency for LeishFuNet.

The data is then split into testing and initial training sets at a ratio of 20:80. The testing set consists of 57 randomly chosen samples, comprising 27 *Leishmania* positive and 30 *Leishmania* negative instances. This random selection ensures unbiased representation in each subset of the data [34]. Meanwhile, the initial training dataset contains 239 images, including 138 *Leishmania* positive and 154 *Leishmania* negative samples. It is crucial to emphasize that the ratio of *Leishmania* positive to negative samples is balanced in both the testing and initial training sets. This approach maintains consistency and fairness during model training and evaluation.

**Fig. 1:**
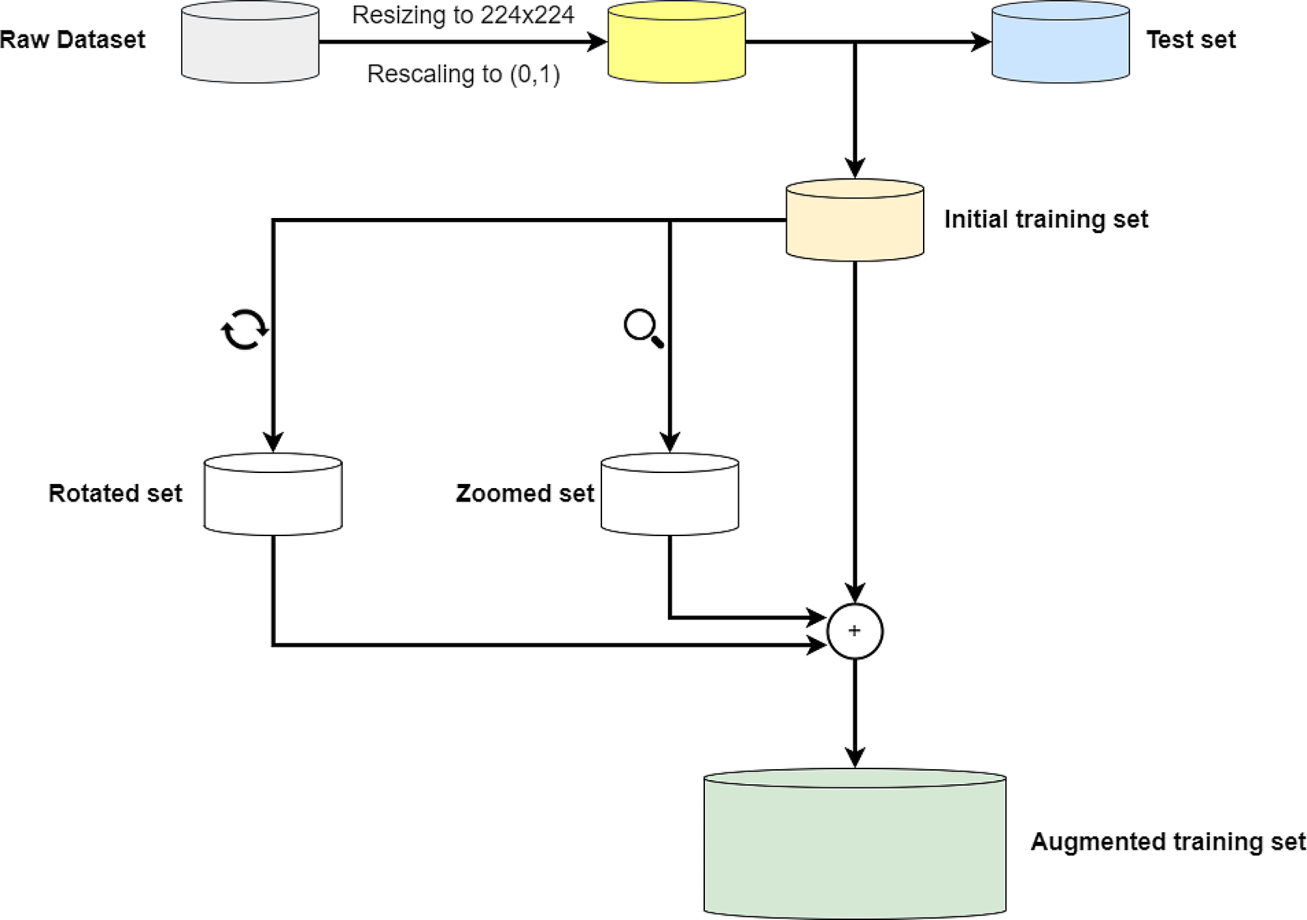
The entire preprocessing pipeline applied to the microscopic images utilized in this study. The initial training set is expanded through random zooming and random rotation of the images within it

To expand the pool of images for training LeishFuNet, we augmented the size of the initial training set using data augmentation techniques. In this process, all samples in the initial training set were randomly rotated up to 0.4π radians in both clockwise and counterclockwise directions, creating a rotated set. Additionally, images in the initial training set were zoomed in and out up to 20% in both the vertical and horizontal directions to generate a zoomed set. By incorporating the rotated and zoomed sets into the initial training set, we created an augmented training dataset, which was utilized for the final training of LeishFuNet. It’s important to note that data augmentation was exclusively applied to the training samples, and the test set remained unaffected by these transformations. Table 1 illustrates the sample counts in each of the aforementioned datasets, while Fig. 1 visually depicts the entire preprocessing pipeline.

**Table 1:** Number of *Leishmania* samples in testing and training datasets. The ratio of *Leishmania* positive samples to *Leishmania* negative samples is equal in both the testing set and training set (both initial and augmented)

	Raw dataset	Testing set	Initial training set	Zoomed set	Rotated set	Augmented training set
*Leishmania* positive	138	27	111	111	111	333
*Leishmania* negative	154	30	124	124	124	372
Total	292	57	235	235	235	705

### Transfer learning

Medical imaging studies often encounter small size datasets and a shortage of data availability, which poses significant challenges for machine learning algorithms. One of the primary reasons for this issue is the strict privacy regulations surrounding medical data, which prohibit the sharing of data between institutions without proper consent [35]. Additionally, accessing medical imaging data can be difficult as it requires secure data systems and specialized medical imaging software [36]. Furthermore, manual annotation of medical images for segmentation and classification can be time-consuming and resource-intensive, further hindering the availability of large datasets [37]. These factors make it difficult for machine learning algorithms to be trained and tested on sufficient data, making it challenging to achieve high-performance results. Therefore, novel techniques such as transfer learning play an important role in addressing the lack of data availability [38].

Transfer learning is the process of leveraging pre-trained models and their learned knowledge from large datasets to solve new tasks with small data size [38]. This is particularly useful in scenarios that gathering large amounts of data to train is challenging, time-consuming or costly (as in medical imaging). Fine-tuning a pre-trained model can save a significant amount of training time and resources without sacrificing performance. This is because the pre-trained models have already learned general features from a vast amount of relevant data, making them a powerful tool in training new models with less data [38, 39]. Transfer learning has been successfully used in various fields, including computer vision [40–42] and natural language processing [43, 44], allowing for the development of more efficient and accurate models.

In this study, we utilized four different pre-trained models: VGG19 [26], ResNet50 [29], MobileNetV2 [45], and DenseNet 169 [46]. These models were originally developed to analyze 224 × 224 RGB images from the ImageNet dataset [47] and classify them into 1000 classes. However, studies have shown that employing pre-trained models trained on same domains can improve transfer learning techniques [48, 49]. To leverage this fact, we first fine-tuned these models on datasets comprising COVID19 negative and positive CT images obtained from [50] and [51] respectively. These datasets are large enough and contain images relevant to COVID19, which shares similarities with leishmaniasis as an infectious disease. By training on such medically relevant data, we aimed to create pre-trained models tailored for medical image analysis tasks, including the classification of *Leishmania* images.

**Fig. 2:**
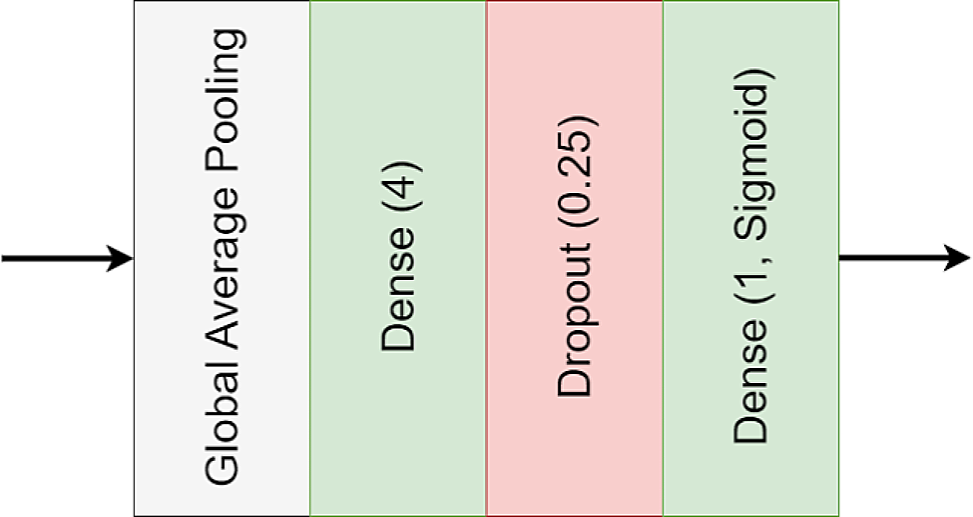
Integration of a new head across four diverse pretrained models, replacing their original heads, for binary classification tasks classifying CT scans into COVID-19 and normal classifications

The training process for the VGG19 [26], ResNet50 [29], MobileNetV2 [45], and DenseNet 169 [46] on COVID19 datasets follows a standardized approach. Initially, we substitute the original classification head of these models, which is designed for classifying objects into 1000 classes, with a new head suitable for binary classification (see Fig. 2). All the layers, including convolutional and dense layers are set to be trainable. Binary cross-entropy serves as the loss function, optimized using the Adam optimizer [52] with a consistent learning rate of 10^− 5^. The training process spans 10 epochs. Hereafter, in this paper, we denote these models trained on COVID-19 data as medical models (medical-VGG19, medical-ResNet50, medical-MobileNetV2, and medical-DenseNet169).

#### LeishFuNet

**Fig. 3:**
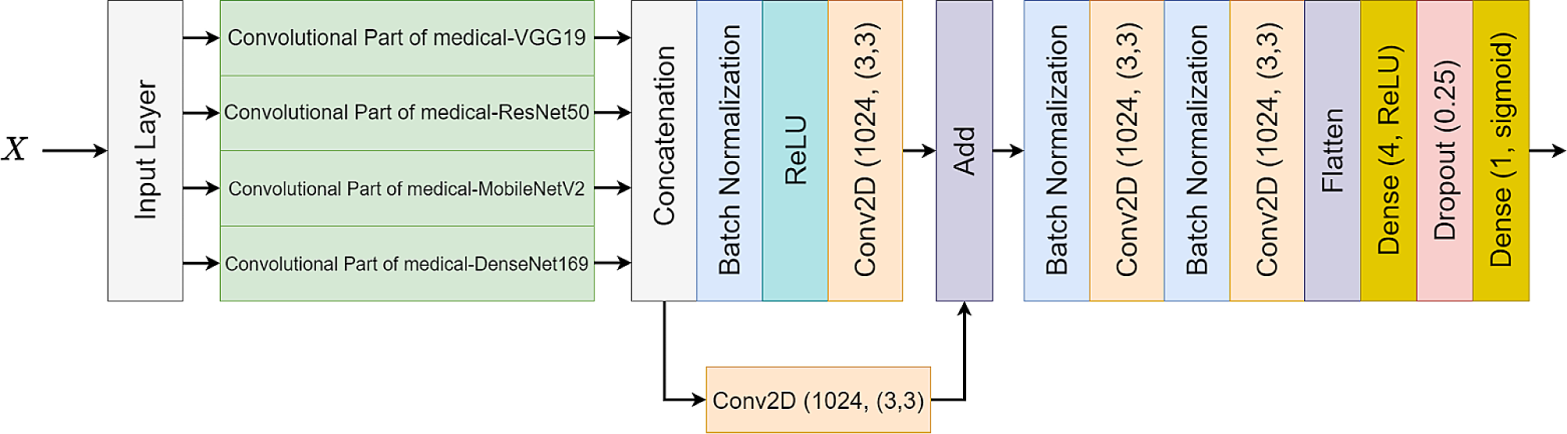
The architecture of LeishFuNet, leveraging the fusion of information extracted by four medical pre-trained models

In this section, we present the architecture of LeishFuNet and its approach to processing input data for detecting *Leishmania* in microscopic images. Figure 3 depicts the general structure of the model, showcasing its utilization of information from four distinct medical pre-trained models. The final prediction is based on the fusion of these analyzed pieces of information. Notably, the head of each medical model is removed, leaving only the convolutional parts frozen to retain the features extracted by each of them.

Let $X \in {\mathbb{R}}^{224\times 224\times 3}$ represent the preprocessed input data to LeishFuNet. Each medical pre-trained model analyzes $X$ and generates a feature vector. These vectors are then concatenated and fed into subsequent layers of LeishFuNet for further analysis.

1 $${H}_{1}= {\mathcal{G}}_{1}\left(X\right), {H}_{2}= {\mathcal{G}}_{2}\left(X\right), {H}_{3}= {\mathcal{G}}_{3}\left(X\right), {H}_{4}= {\mathcal{G}}_{4}\left(X\right),$$

2 $${H}_{fu}=\left({H}_{1} \right|\left| {H}_{2} \right|\left| {H}_{3} \right|\left|{ H}_{4}\right)$$

Where ${\mathcal{G}}_{1}$, ${\mathcal{G}}_{2}, {\mathcal{G}}_{3}, {\mathcal{G}}_{4}$ denote the functions of the convolutional part of the medical-VGG19, medical-ResNet50, medical-MobileNetV2, and medical-DenseNet169 models, respectively. Correspondingly ${H}_{1}\in {\mathbb{R}}^{7\times 7\times 512}$, ${H}_{2}\in {\mathbb{R}}^{7\times 7\times 2048}$, ${H}_{3}\in {\mathbb{R}}^{7\times 7\times 1280}$, ${H}_{4}\in {\mathbb{R}}^{7\times 7\times 1664}$ represent the output vectors of these models containing the extracted features from the input data analysis. The concatenation operator, $\left|\right|$, combines these feature vectors, resulting in ${H}_{fu}\in {\mathbb{R}}^{7\times 7\times 5504}$ which is a single vector composed of the concatenated features extracted from the medical pre-trained models.

Upon concatenating the extracted features, ${H}_{fu}$proceeds through two distinct pathways. In the first pathway, the ReLU activation function is applied alongside a convolutional layer to enhance the model’s non-linearity. In contrast, the second pathway employs only a convolutional layer to prevent potential data loss caused by the ReLU activation function [53]. This ensures uninterrupted flow of data to subsequent layers of LeishFuNet, as depicted in Fig. 3.

To address over fitting, we integrated a dropout layer with a drop rate of 0.25 into our model. This layer systematically removes 25% of neurons from its preceding layer during each step of the training process (one neuron at a time within LeishFuNet). By introducing randomness, dropout enhances the model’s resilience to new, unseen data and reduces the risk of over fitting [54]. Importantly, this dropout layer is active solely during the training phase; during testing, it does not alter the model’s architecture. Finally, aided by a single neuron with a sigmoid activation function, the model performs the final classification, categorizing microscopic images into two groups: *leishmania*-infected and non-infected.

## Results

### Training process

In this section, the focus will be on providing an explanation of the hyperparameters chosen and optimization techniques used during the training process of the model. Hyperparameters play an essential role in determining the accuracy and efficiency of a trained model, and selecting the right values for hyperparameters is critical [55].

In this study, the selection of the loss function was based on the consideration that the objective was binary classification, wherein each sample was classified into one of two categories. Accordingly, the binary cross entropy was determined as loss function. Additionally, multiple optimization methods were evaluated, including Adam [52], RMSProp [56], and SGD, to determine the optimizer that would achieve the best performance. The results indicated that the model performed the best while utilizing Adam, thus it was selected as the final optimizer for the training process. The model was trained over 40 epochs with a batch size of 32. The entire training process was conducted using TensorFlow [57] on a Kaggle kernel with a GPU100 accelerator.

The training process follows a 5-fold cross-validation methodology, a widely recognized technique in machine learning employed to assess both the accuracy and generalization performance of a model. In this method, the original dataset is divided into five equal-sized subsamples, or folds. Four of these folds are used for training the model, while the remaining fold is used for testing the model. This process is repeated for each of the five folds, with each fold being used as the test data once. By utilizing different folds of the data for training and testing, the model is exposed to a variety of test datasets and this helps to ensure that the model is not over fitting. In addition, 5-fold cross validation can help in selecting the best model from a set of models. By evaluating each model on different folds of the data, the performance of each model can be easily compared and most optimal model will be selected.

The entire training process incorporates a learning rate scheduler. Initially, the learning rate is set to 10^− 3^ and gradually decreases as training progresses, culminating in a value of 10^− 5^ for the final epoch of training.

### Assessment metrics

A deep learning model’s performance can be evaluated by several metrics depending on the objective of the model. For image classification tasks, such as our study, some commonly used performance evaluation metrics include: accuracy, precision, recall or sensitivity, specificity, and f1-score. There are 4 terms by using which these metrics calculate a model’s performance. These terms are:

- True Positive (TP): positive samples classified correctly by model.
- True Negative (TN): Negative samples classified correctly by model.
- False Positive (FP): Negative samples classified incorrectly as positive by model.
- False Negative (FN) Positive samples classified incorrectly as negative by model.

With these terms in hand, metrics are described below:

3 $$Accuracy= \frac{TP+TN}{TP+TN+FP+FN}$$

4 $$Precision= \frac{TP}{TP+FP}$$

5 $$Recall\,or\,Sensitivity= \frac{TP}{TP+FN}$$

6 $$Specificity= \frac{TN}{TN+FP}$$

7 $$F1-Score= \frac{2\times Precision \times Recall }{Precision+ Recall }$$

Apart from these metrics, Area Under the Curve (AUC) is another metric commonly used in machine learning and binary classification problems to measure how well a model can distinguish between positive and negative classes. It is represented as a value between 0 and 1, where 0.5 means the model is not effective at distinguishing between the positive and negative classes, and 1 means it is perfect. The higher the AUC value, the better the model’s performance.

#### LeishFuNet performance

We developed LeishFuNet using the augmented training set generated during the preprocessing steps (see Sect. 2.2). Initially, the augmented training set is divided into five equal-sized folds in a stratified manner, ensuring that the number of positive and negative samples remains consistent across all folds. Subsequently, four of these folds are used for training the model, while the remaining fold serves as the validation set for hyper parameter fine-tuning. Table 2 provides a breakdown of the *Leishmania* positive and negative samples in each fold.

**Table 2:** Distribution of *Leishmania* positive and negative samples in each fold

	Number of samples				
	Fold 1	Fold 2	Fold 3	Fold 4	Fold 5
Leishmania positive	66	66	67	67	67
Leishmania negative	75	75	74	74	74
Total	141	141	141	141	141

**Fig. 4:**
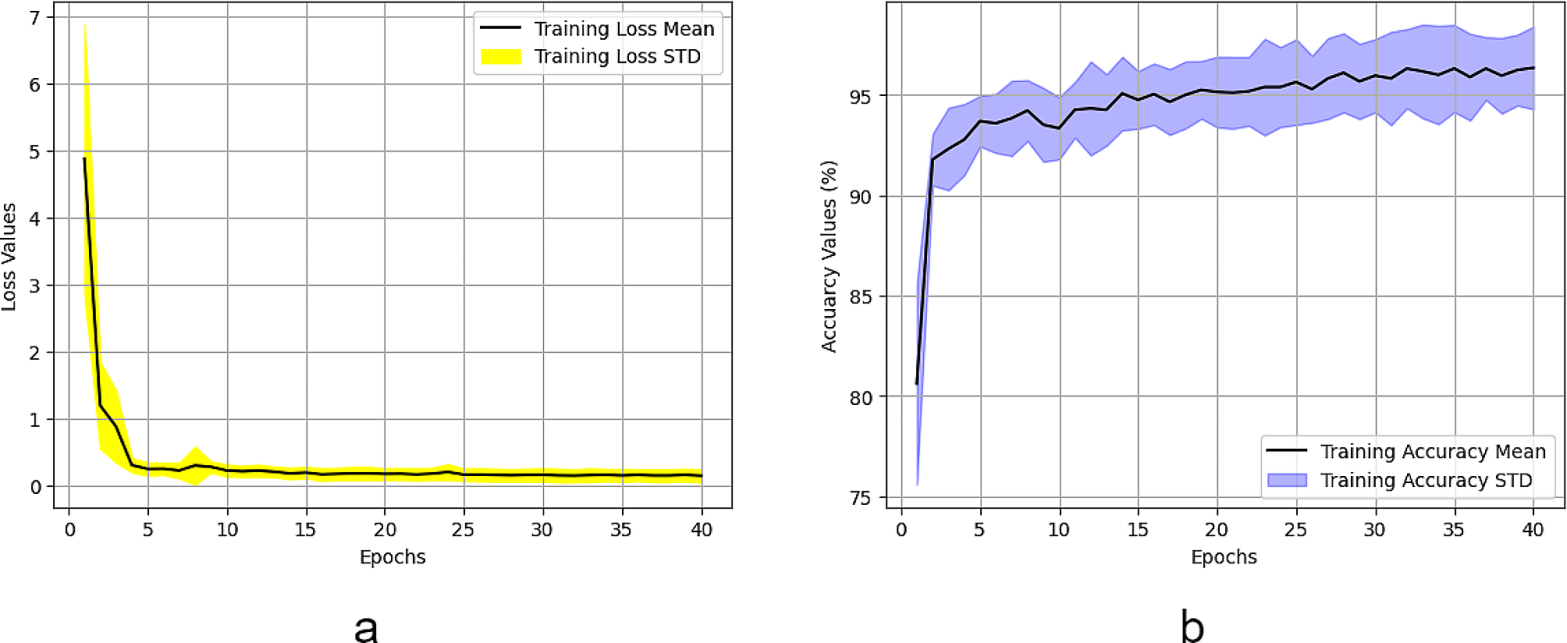
Performance of LeishFuNet during training. (**a**) Shows changes in loss values, while (**b**) illustrates changes in accuracy values throughout the training process

Five different models are developed training on five distinct training sets, and Table 3 displays their performance on the testing set. Despite being developed on various variations of the training data, the mean and standard deviation values for these models indicate consistent and high performance. Furthermore, Fig. 4 illustrates the continuous improvement and convergence to an optimal value of both accuracy and loss values during the training process.

**Table 3:** The performance of different developed LeishFuNet

	Accuracy (%)	Precision (%)	F1 score (%)	Specificity (%)	Sensitivity (%)	AUC (%)
Fold 1	98.25	96.43	98.18	96.67	100	98.33
Fold 2	96.49	93.1	96.43	93.33	100	96.67
Fold 3	100	100	100	100	100	100
Fold 4	100	100	100	100	100	100
Fold 5	100	100	100	100	100	100
Average	98.95 $\pm$ 1.4	97.91 $\pm$ 2.77	98.92 $\pm$ 1.43	98 $\pm$ 2.67	100	99 $\pm$ 1.33

### Grad – CAM on leishfunet

Deep learning models, with their complex architectures, often lack transparency in how they arrive at decisions, making it difficult for clinicians to trust and understand their outputs. Explainable artificial intelligence (XAI) techniques are imperative in this context, offering insights into model decisions and enhancing interpretability. XAI methods such as Grad-CAM [58] provide clinicians with understandable rationales behind predictions, enabling them to validate model outputs, improve trust, and ultimately facilitate the integration of deep learning models into clinical practice while ensuring patient safety and care quality.

Grad-CAM, short for Gradient-weighted Class Activation Mapping, is a technique used in deep learning for visualizing and understanding which regions of an image are important for predicting a particular class. It generates heatmap visualizations by examining the gradients of any target class flowing into a convolutional layer of a CNN. By highlighting these important regions, Grad-CAM provides insights into the decision-making process of the model, aiding researchers and practitioners in interpreting and debugging deep learning models, particularly in tasks like object detection and image classification.

**Fig. 5:**
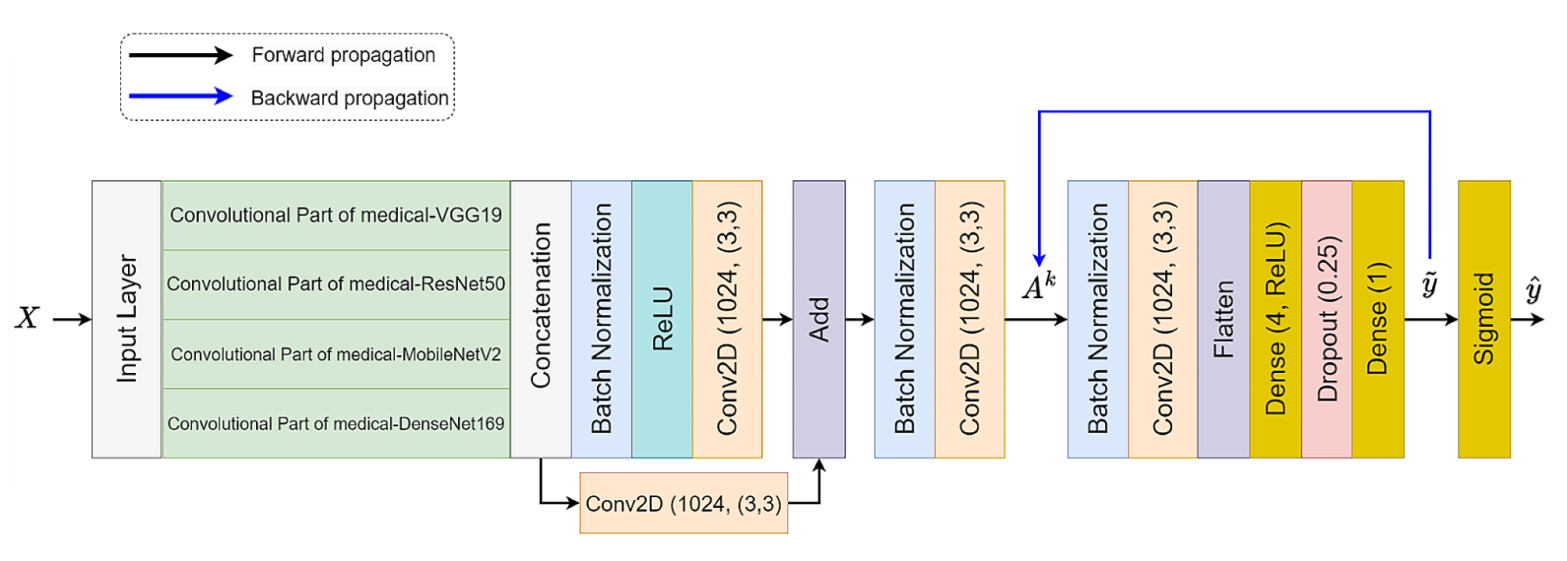
Implementing Grad-CAM on LeishFuNet involves calculating gradients of LeishFuNet’s output score with respect to the output of the second-to-last convolutional layer

In this study, for implementing Grad-CAM on LeishFuNet for binary image classification, the first step involved computing the gradient of the score produced by the last dense layer (before the sigmoid function) with respect to the output feature map of the second-to-last convolutional layer within LeishFuNet ($\frac{\partial \stackrel{\sim}{y}}{\partial {A}^{k}}$, where $\stackrel{\sim}{y}$ is the score and ${A}^{k}\in {\mathbb{R}}^{3\times 3\times 1024}$ is the feature map generated by the second-to-last convolutional layer (see Fig. 5)). Next, by implementing global average pooling, the importance weight of each feature map is calculated.

8 $${\alpha }_{k} = GAP\left(\frac{\partial \stackrel{\sim}{y}}{\partial {A}^{k}}\right)$$

${\alpha }_{k} \in {\mathbb{R}}^{1024}$ is the importance weight of feature maps and $GAP$ represents the global average pooling operator. By multiplying these importance weights with their corresponding feature maps, a heat map for each image is generated. It is noteworthy that, unlike the original Grad-CAM approach where ReLU was applied to the weighted combination of feature maps to retain only the positive values, in our implementation, we omitted the ReLU activation to preserve all values contributing to LeishFuNet’s performance. This decision was made to maintain the integrity of all information that influences the model’s performance.

**Fig. 6:**
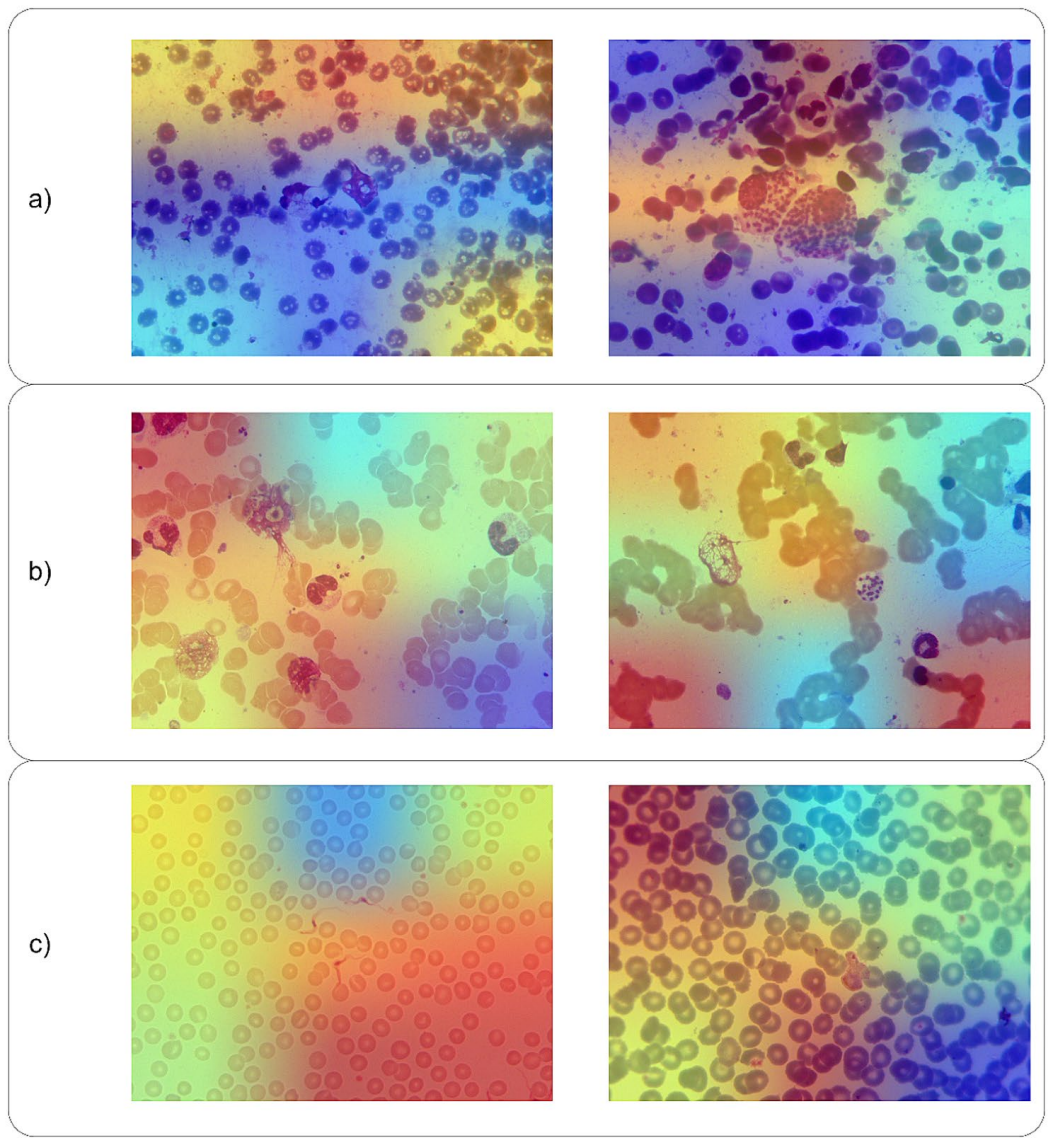
**a**) Analyzed regions of the microscopic images by LeishFuNet, leading the model to classify these images as *Leishmania* positive. **b**) Analyzed regions of low-density *Leishmania* parasite images by LeishFuNet for prediction. **c**) Analyzed regions of negative *Leishmania* parasite images by LeishFuNet for prediction

Analysis of the heat maps generated by Grad-CAM on test data predicted by LeishFuNet reveals that the model processes microscopic images in a manner consistent with clinical observation. Specifically, when the model searches for *Leishmania* parasites within medical images and detects them in certain sections of the microscopic image, it assigns a positive label to the image. Figure 6.a illustrates the specific areas within each image analyzed by LeishFuNet for predicting *Leishmania* -positive images.

When the *Leishmania* parasite density in a microscopic image is high, the model readily detects these parasites, often requiring analysis of only a few regions to classify the image as *Leishmania* positive. To evaluate the model’s robustness in scenarios with low-density *Leishmania* parasites, we generated heatmaps using Grad-CAM for such images. As depicted in Fig. 6.b, compared to the high-density *Leishmania* images shown in Fig. 6.a, the model scrutinizes more regions to make predictions. This observation underscores the model’s robust performance when handling images with low *Leishmania* parasite densities.

Upon examining the heatmaps generated by LeishFuNet for *Leishmania* -negative images, it is evident that the model searches for *Leishmania* parasites across almost all regions of the image. If none of the analyzed regions indicate the presence of *Leishmania* parasites, the image is classified as negative (see Fig. 6.c).

The findings from implementing Grad-CAM on LeishFuNet demonstrate that the proposed model operates similarly to medical experts when analyzing microscopic images to detect *Leishmania* parasites. This similarity underscores the trustworthiness of LeishFuNet and its potential utility in practical applications.

### LeishFuNet performance on another dataset

To demonstrate that LeishFuNet’s performance on our newly introduced dataset was not merely coincidental and that its effectiveness extends beyond this specific dataset, we conducted an evaluation using a different *Leishmania* dataset. For this purpose, we utilized the Microscopic Images of Parasites Species [59], which includes images of various parasites including *Leishmania*. This dataset was compiled by dividing microscopic images for each patient into multiple fragments, with each fragment containing the image of a single parasite. Table 4 presents the types of parasites included in this dataset along with the corresponding quantities of each parasite. To prepare this dataset for binary classification, which is appropriate for evaluating our model, we specifically chose all the *Leishmania* images (2701) and *Trypanosome* images (2385). *Trypanosome* images were selected because they exhibited the closest visual similarity to *Leishmania* images among all the available microscopic images of parasites. We applied the same preprocessing steps used previously, resizing all images to dimensions of 224$\times$224 and rescaling their pixel values to a range between 0 and 1. Subsequently, we randomly selected 4000 images (78.65%) for training purposes, while 1086 samples (21.35%) were set aside for the testing phase. Table 5 provides detailed information regarding the number of parasites utilized for developing LeishFuNet.

**Table 4:** Parasite species and corresponding image counts in the microscopic images of parasites species dataset

	Babesia	Leishmania	Leukocyte	Plasmodium	RBC	Toxoplasma	Trichomonad	Trypanosome
# of images	1173	2701	1376	843	8995	6691	10,134	2385

**Table 5:** Detailed information on the number of parasites utilized for developing LeishFuNet.

	Number of images	
	Train	Test
*Leishmania*	2117	584
*Trypanosome*	1883	502
Total	4000	1086

**Fig. 7:**
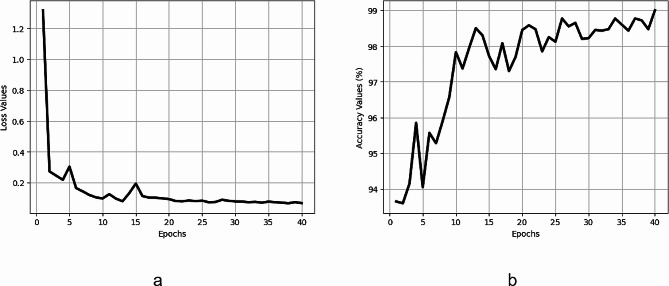
Changes in **a**) loss values and **b**) accuracy during the training of LeishFuNet on the microscopic images of parasites species dataset

Hyper parameter tuning steps were not implemented, and we opted to use the same hyper parameters as those employed previously in the development of LeishFuNet. The optimizer chosen was Adam, and binary cross-entropy served as the loss function. Throughout the training process, the learning rate gradually adjusted from 10^− 3^ to 10^− 5^. Training was conducted over 40 epochs. We did not utilize five-fold cross validation as our objective was not to fine-tune the model, but rather to evaluate its performance. According to Table 6, which presents the performance of LeishFuNet on the microscopic images of parasites species dataset, it is evident that our proposed model effectively detects *Leishmania* parasites within this dataset. Additionally, Fig. 7 illustrates the fluctuations in accuracy and loss function throughout the training process, confirming that the model appropriately converges to an optimal value.

**Table 6:** Performance of LeishFuNet in detecting *Leishmania* parasites in the microscopic images of parasites species dataset

	Accuracy	Precision	F1 score	Specificity	Sensitivity	AUC
LeishFuNet performance	99.91%	100%	99.91%	100%	99.83%	99.91%

## Discussion

Deep learning techniques are widely employed in various medical studies, including research on parasite detection. Access to a suitable dataset is crucial for developing such models. However, a major limitation in developing deep learning models for *Leishmania* detection is the lack of accessible public data. This study aims to address this issue by providing a clean and publicly accessible dataset containing microscopic images from both *Leishmania* -infected and non-infected patients. This dataset can significantly contribute to the development of advanced deep learning models by researchers in this field, facilitating easier detection of the disease and helping in its prevention and control.

Many studies in this field have developed their models using private datasets, making it challenging to evaluate LeishFuNet on their datasets and to provide a fair comparison of our model’s performance with other deep learning models. Consequently, we compared our study with existing research using other tangible criteria.

Firstly, LeishFuNet is designed for patient-level detection, whereas previous studies focused on slide-level detection. In slide-level detection, the model categorizes slides into positive and negative samples. In such studies, each patient may contribute multiple slides to the training data, potentially biasing the model towards certain hidden features specific to particular patients rather than features from the microscopic images themselves. Moreover, it’s possible that slides from the same patient are present in both the training and testing sets, which can undermine the generalizability and reliability of the model’s performance. In contrast, LeishFuNet is trained using microscopic images, each derived from a separate patient. Therefore, it does not encounter these issues, and its reported performance is deemed reliable.

Secondly, LeishFuNet stands out as an end-to-end model, unlike several previous studies in the field of *Leishmania* detection. Many of these studies utilized machine learning techniques that require specialists for feature extraction during both the training and testing phases. This issue is circumvented in end-to-end models like LeishFuNet.

Another notable improvement over previous studies in our research is the utilization of XAI techniques. In healthcare-related studies where decision-making is critical, deploying deep learning models in practical applications necessitates demonstrating their trustworthiness [60]. While previous studies demonstrated high performance in detecting *Leishmania*, they did not incorporate XAI techniques to validate the trustworthiness of their models for practical use. In contrast, LeishFuNet, with the assistance of Grad-CAM, demonstrated that it classified microscopic images in a manner similar to clinicians. The implementation of Grad-CAM on LeishFuNet revealed that the model searches for *Leishmania* parasites within each input image and if it detects a few parasites, the image is classified as *Leishmania* positive. It has been observed that in densely infected images, the model classifies the image by focusing on limited regions, whereas in less densely infected images, it investigates a wider region. For *Leishmania* negative samples, the model only classifies them as non-infected if it thoroughly examines all regions of the image and finds no evidence of the parasite’s existence. This process mirrors the approach taken by clinicians in identifying *Leishmania* -infected patients from their microscopic images. This underscores that LeishFuNet not only achieves high performance but also does so with a systematic approach, demonstrating its trustworthiness for practical applications. Moreover, alongside Grad-CAM, other XAI techniques like LIME [61], SHAP [62], and RISE [63] can be leveraged in future studies to analyze the model’s prediction process and further enhance its trustworthiness.

To quantitatively compare our proposed model with state-of-the-art deep learning models, we evaluated MobileViT [64], an advanced model that combines convolutional neural networks and visual transformers, on our introduced dataset. We then compared its performance with LeishFuNet. The results, summarized in Table 7, indicate that LeishFuNet outperformed MobileViT in most metrics. This suggests that LeishFuNet is more suitable for detecting *Leishmania* compared to powerful deep learning models like MobileViT.

**Table 7:** Performance of MobileViT and LeishFuNet on the introduced *Leishmania* dataset. These results demonstrate that LeishFuNet surpasses powerful and state-of-the-art deep learning models in detecting *Leishmania* in microscopic images

	Accuracy (%)	Precision (%)	F1 score (%)	Specificity (%)	Sensitivity (%)	AUC (%)
MobileViT	96.49	**100**	96.15	**100**	92.59	0.963
LeishFuNet	**98.95**	97.91	**98.92**	98	**100**	**99**

Despite the mentioned advantages of LeishFuNet outlined above, this study also has several limitations:

- **Small Dataset Size**: The dataset used to develop LeishFuNet is relatively small. While we addressed this issue by employing transfer learning techniques, developing future deep learning models using larger datasets of *Leishmania* microscopic images could yield improved results.
- **Limited to a Single Microscope Type**: LeishFuNet is trained exclusively on a single type of microscopic images captured using the Olympus-CX23-TR-Japan microscope. Expanding the scope of future deep learning models to include images from various types of microscopes can mitigate potential biases in the results and enhance the reliability of the model.

## Conclusion

In this research, we introduced LeishFuNet, an end-to-end model designed to detect *Leishmania* at the patient level in microscopic images. This model eliminates the need for expert analysis of such images, thereby reducing the additional costs associated with CL diagnosis. Leveraging feature fusion and transfer learning techniques, LeishFuNet has demonstrated superior performance compared to state-of-the-art deep learning models in detecting *Leishmania*. Additionally, it has exhibited high performance on a separate public dataset, showcasing its generalizability and ability to accurately process new, unseen samples. Through the utilization of Grad-CAM, we illustrate that LeishFuNet’s decision-making process closely resembles that of human clinicians, enhancing its reliability and applicability in real-world scenarios. Our solution offers an economical alternative for the examination of CL. Also, we introduce a publicly available dataset of leishmaniasis microscopic images comprising both positive and negative samples in a roughly equal proportion. This dataset is valuable for further research using deep learning and machine learning techniques in the field of leishmaniasis.

## Data Availability

The data that support the findings of this study are available from the corresponding author upon reasonable request. Furthermore, the developed codes and the dataset implemented in this study are available via: https://github.com/alrzsdgh/leishmania.

## Abbreviations

CL: Cutaneous Leishmaniasis
NTD: Neglected Tropical Diseases
MCL: Mucocutaneous Leishmaniasis
VL: Visceral Leishmaniasis
PKDL: Post-Kala-azar Dermal Leishmaniasis
CNN: Convolutional Neural Network
SGD: Stochastic Gradient Descent
RMSProp: Root Mean Square Propagation
Adam: Adaptive Moment estimation
SVM: Support Vector Machine
KNN: K-Nearest Neighbors
XAI: eXplainable Artificial Intelligence
Grad-CAM: Gradient-weighted Class Activation Mapping
